# A scoping review of research capability building: impact on health workforce attraction and retention in rural and remote Australia

**DOI:** 10.1186/s12960-026-01069-9

**Published:** 2026-04-25

**Authors:** Tsegaye G. Haile, Justin Manuel, Mohamed Estai

**Affiliations:** 1 Curtin School of Population Health, Curtin University, Perth, WA 6102 Australia; 2 Department of Health Systems and Policy, Institute of Public Health, University of Gondar, Gondar, Ethiopia; 3 WA Country Health Service, Bentley, WA 6102 Australia

**Keywords:** Research capacity building, Health workforce outcomes, Attraction and retention, Rural and remote settings, Australia

## Abstract

**Background:**

Research capacity building (RCB) aims to enhance skills, embed evidence‐informed practice, and contribute to improving patient and workforce outcomes, particularly in rural and remote areas that are disproportionately affected by workforce shortages, limited resources, and geographic isolation. However, evidence on the impact of RCB on health workforce outcomes and the barriers and enablers influencing RCB implementation and its sustainability remains limited. Thus, we mapped the available evidence on the relationship between RCB and health workforce outcomes, including attraction and retention in rural and remote areas, identified key success factors and challenges, and highlighted strategies to inform future policy and practice.

**Methods:**

A scoping review of published and publicly available grey literature from Australia (January 2000 to end of February 2025) was undertaken following the PRISMA–ScR 2020 statement and Joanna Briggs Institute methodology. CINAHL, MEDLINE, ProQuest Central, and Scopus online databases were searched, supplemented by Google, Google Scholar, and reference snowballing. The interconnectedness of the following domains was explored: (i) health professionals, (ii) research engagement and RCBs, (iii) workforce outcomes, such as attraction and retention, and (iv) rural/remote settings. Both qualitative and quantitative studies were included, with descriptive and thematic analyses conducted deductively and inductively following the socioecological model.

**Results:**

Nineteen studies were included: nine qualitative, six quantitative, and four mixed methods. Three examined RCB implementations, three assessed the impact of RCB, one evaluated program effectiveness, and the remainder investigated multiple outcomes, including barriers and facilitators. Included studies reported that RCB initiatives were described as contributing to improved research skills, increased professional satisfaction, and perceived workforce retention, particularly when initiatives provided mentorship, protected time, and addressed locally relevant priorities. Common barriers identified limited organisational support, high workloads, and short‐term funding, while strong leadership, embedded research facilitators, and alignment with community needs were key enablers. Longitudinal evidence directly linking RCB initiatives to measurable improvements in workforce attraction and retention was scarce.

**Conclusions:**

Our findings suggest a potential association between RCB and strengthened skills, increased professional satisfaction, and improved workforce stability in rural and remote areas. To sustain the impacts of RCB, the findings highlight the importance of locally relevant design, ensure adequate resourcing, and provide strong organisational leadership support. Finally, robust longitudinal evaluations of the impact of RCB on workforce attraction and retention will be essential to confirm and optimise its potential benefits.

**Supplementary Information:**

The online version contains supplementary material available at 10.1186/s12960-026-01069-9.

## Introduction

Research capacity building (RCB) is defined as the process of strengthening long-term skills and competencies of individuals and institutions that enable to undertake robust and high-quality research activities [1]. RCB is considered as a key strategy for improving the health system’s quality, efficiency, and equity [2]. This strategy is particularly critical in rural and remote healthcare settings, where services are constrained by structural limitations, workforce shortages, and limited access to context-specific data [3, 4].

In Australia, with chronic health workforce shortages in rural and remote areas, persistent challenges in accessing timely and appropriate healthcare are contributing to poorer outcomes and higher healthcare costs [5–7]. For example, in 2024, the full-time equivalent (FTE) rate of clinical health professionals in remote and very remote areas was 1938 and 1846 per 100,000 population, respectively, compared with 2248 per 100,000 in major cities [8]. This workforce shortage in remote regions reduced access to health services, delayed care, and increased a disproportionate burden of chronic disease, higher preventable hospitalisations rates, and unmet health needs [9]. In a similar report, the overall burden of disease in remote areas is 1.4 times that of major cities, with 243.9 disability adjusted life years (DALYs) per 1000 population and 173.7 DALYs per 1000 population, respectively. Evidence suggested that besides the limited health workforce availability and high staff turnover, geographic isolation also contributed to the disproportionate healthcare burden in rural and remote areas [10], which was also observed in other underserved settings globally [5]. This underscores the need to address health workforce shortages and maldistribution, as well as to strengthen workforce capacity to enhance individual knowledge and skills and contribute to improve health services and workforce outcomes.

RCB could enable healthcare professionals to improve health outcomes, identify local challenges in rural and remote areas, and potentially contribute to better service delivery [11] and support professional engagement [12]. Research engagement, often fostered through RCB, is increasingly recognised as a promising strategy for improving workforce outcomes in rural and remote Australia [13, 14]. This workforce outcome improvement might be through improved job satisfaction [15], professional development [16], and organisational support [17], factors that strongly influence workforce attraction and retention. However, sustaining RCB in rural and remote Australia is constrained by systemic, organisational, and individual-level barriers. At a structural level, fragmented collaborations between academic institutions and health services [13, 18], limited research infrastructure [11, 14, 18, 19], and reliance on short-term project funding undermine long-term investment and continuity. Organisational challenges, such as high staff turnover [14, 20, 21], heavy clinical workloads [13, 15, 18], and the absence of protected time for research [16, 22], further restrict the ability of rural health services to prioritise or embed research activities. From an individual perspective, clinicians in rural areas frequently cite limited access to mentorship, insufficient training, and a lack of confidence or experience in research methods as key constraints to participation [11, 18, 19]. These barriers collectively contribute to a disconnection between academic research and rural health service delivery realities, limiting opportunities for locally driven innovation and the integration of evidence into practice [23].

In response to ongoing research capacity gaps in rural and remote Australia, various RCB initiatives have been developed as a strategic approach to support local health professionals and services [24–26]. These are intended to enhance individual research skills, foster team and/organisational readiness, and embed research into everyday healthcare practice at the system level [27]. The structure and focus of RCBs differ across states and territories, including models, such as formal training programs, collaborative academic-health service initiatives, regionally embedded research roles, and practice-based research networks. In Australia, several state governments have established Rural Research Capacity-Building programs (RRCBPs), including the Health Education and Training Institute (HETI) in New South Wales (NSW) [25, 26], the Supporting Translation of Research in Rural and Regional settings (STaRR) Program in Victoria [28, 29], and the Rural and Remote Research Capacity-Building Program (RRR-Cap) in Queensland [30]. These initiatives are designed to promote clinician-led research, provide funding for dedicated research roles and create regional hubs that drive research addressing local health priorities. While these programs reflect a growing commitment to rural research development, questions remain about their long-term impact on workforce stability, specifically attraction and retentions, ability to adapt to diverse community, and service needs in the rural and remote areas.

Despite the growing number of RRCBPs in Australia, the depth and breadth of evidence on RCB, its contribution to health workforce outcomes, factors influencing implementation success and failure, and associated sustainability challenges, remain unknown [26, 27]. Challenges such as the underutilisation of local clinician expertise, misalignment with local health service priorities, and limited coordination between States and Territories may have hindered the effectiveness of existing RCB initiatives. Addressing these evidence gaps is both timely and essential, aligning with national priorities, such as the Medical Research Future Fund (MRFF), Closing the Gap reforms, and the National Rural Health Strategy [31, 32]. Therefore, our scoping review aimed to map the available evidence on RCB and its association with health workforce outcomes in rural and remote areas, particularly attraction and retention, while identifying the key facilitators, barriers, and effective strategies of RCB implementation to inform sustainable and locally led research efforts that can contribute to strengthen rural health systems and reduce persistent inequities. In this scoping review, we addressed five key research questions:

1. What evidence exists on the relationship between RCB and health workforce outcomes, such as attraction, retention, and stability, in rural and remote areas?
2. What is the impact of RCB initiatives on health workforce outcomes across multiple levels?
3. What factors facilitate the implementation and sustainability of RCB to improve workforce outcomes?
4. What barriers limit the implementation, reach, and impact of RCB in rural and remote health areas?
5. What strategies have been identified to optimise RCB implementation for strengthening workforce outcomes, including attraction, retention, and workforce stability?

## Methods

### Review design

We employed a scoping review. A scoping review provides a preliminary assessment of the potential size and scope of the available research literature, aiming to identify the nature and extent of evidence on RCB and its contribution to health workforce outcomes [33] (Supplementary Table S1). This approach was appropriate for mapping key concepts, identifying evidence gaps, and characterising the breadth of existing research. Our scoping review followed the Joanna Briggs Institute (JBI) methodology for scoping reviews [34] and adhered to the Preferred Reporting Items for Systematic Reviews and Meta-Analyses extension for Scoping Reviews (PRISMA–ScR) 2020 checklist [35]. Furthermore, this scoping review was conducted following a methodological framework and all the detailed methods were published [36].

### Data sources and search strategy

A comprehensive search strategy was developed following the PRISMA–ScR guidelines and the PCC (participants, concept, context) framework [37], as outlined in our study protocol [36]. We searched five major electronic databases, including CINAHL (EBSCO), MEDLINE (Ovid), Scopus, and ProQuest Central to identify peer-reviewed literature. In addition, we searched Google and Google Scholar for publicly available grey literature, including program evaluations, project/program reports, and other non-indexed sources relevant to rural and remote health workforce initiatives. To ensure comprehensive coverage, citation snowballing was conducted by manually screening the reference lists of all included studies. In this process, we identified additional eligible studies not captured through initial database searches.

The PCC framework guided the development of our search terms. Participants included all types of healthcare professionals, including the four broad categories, nurses and midwives, medical practitioners, dental practitioners, and allied health professionals, defined by the Australian Institute of Health and Welfare (AIHW) [8]; Concept was referred to as engagement in RCB programs, strategies, or initiatives (formal and informal opportunities to enhance research literacy, skills, and leadership); Context was about settings, such as regional, rural, remote, underserved, disadvantaged, or hard-to-reach areas of Australia. These terms were included to capture the broad terminology used to describe non-metropolitan healthcare settings.

Search strategies were tailored to each database using keywords and subject headings with a relevant Boolean operator (OR/AND). Terms broadly encompassed four key domains: (i) health workforce, (ii) research capacity building, (iii) workforce outcomes, such as attraction, retention, and stability, and (iv) rural and remote settings in Australia. The complete search strategy for each database is detailed in Supplementary Table S2. Our searches included the period between January 1, 2000, and February 25, 2025.

### Study selection

As outlined in our published protocol [36], we used a two-stage screening process. All records retrieved from the comprehensive database and grey literature searches were imported into Covidence™. Duplicates were removed automatically and were manually verified. In the first screening stage, one reviewer (TGH) assessed the titles and abstracts of all records against the inclusion criteria. A second reviewer (ME) independently screened a random 25% of the titles and abstracts, and resolved records labelled “maybe” by the first reviewer. Discrepancies were discussed and resolved by consensus.

In the second stage, two reviewers (TGH and ME) independently assessed the full texts of all potentially eligible studies. Disagreements regarding inclusion were resolved through weekly discussions. Citation tracking (backward and forward snowballing) was conducted by one reviewer (ME) and independently checked by the second reviewer (TGH). Finally, a consensus discussion was held between the two reviewers to confirm the final set of included studies.

#### Eligibility criteria

We included all empirical primary studies and program reports published between January 1, 2000, and February 25, 2025, using qualitative, quantitative, or mixed-methods designs as well as relevant grey literature, such as evaluation or project reports. Studies which reported on RCB among rural health professionals in Australia and its relationship to workforce outcomes such as attraction, retention, and sustainability were included. We excluded systematic reviews (though their references were scanned), studies focused exclusively on urban/metropolitan settings, and opinion/commentary articles. No language limits were applied; however, all retrieved studies were published in English. Full inclusion/exclusion criteria are detailed in the published protocol [36] and Supplementary Table S3.

#### Data extraction

A comprehensive data extraction framework was developed in alignment with the study protocol [36]. The initial version of the extraction tool was designed in Microsoft Excel™ and piloted independently by two reviewers (TGH and ME) on a sample of five included studies to assess clarity, comprehensiveness, and inter-rater reliability. Based on this pilot, the tool was refined to ensure it captured all relevant data dimensions across study types and data sources. This included fields to document variations in study design, research engagement models, context, and workforce outcomes. Following the pilot, the two reviewers (TGH and ME) extracted data from all included studies. They then met weekly, after every five studies were extracted, to verify the accuracy and completeness of the data, ensure consistency, and resolve any ambiguities. The final data extraction matrix covered both peer-reviewed studies and publicly available grey literature and included the following domains: (1) study characteristics; (2) participant characteristics; (3) characteristics of RCB, such as type and scope of engagement (e.g., research training, collaborative partnerships, embedded researcher models, and community-based participatory research) and delivery mechanisms; (4) implementation factors, such as enabling factors, barriers, leadership involvement, mentorship, partnerships, infrastructure support, and cultural or organisational alignment; (5) health workforce outcomes, such as attraction, retention, professional satisfaction, workforce stability, turnover, or documented intentions to remain; and (6) best strategies: what works for better implementation of the program/initiatives. A separate but aligned framework was used to extract information from both qualitative and quantitative methods.

#### Data synthesis

Our data synthesis followed a rigorous, iterative process combining thematic analysis, framework synthesis, and narrative integration. The analytical approach was both deductive, drawing from the SEM [38, 39], as illustrated in the published protocol [36], and inductive, allowing for emergent themes not captured by existing frameworks.

First, we provided a narrative description of the available evidence, followed by a detailed overview of RCB. Second, we then examined the impact of RCB on health workforce outcomes at the individual, organisational, and system levels, using the SEM to guide our interpretation. Third, we synthesised findings on the role of RCB as a workforce attraction and retention strategy, highlighting its contributions to strengthening rural and remote health workforce stability. We then identified the key enablers and barriers related to RCB implementation and, finally, synthesised the best strategies to support improved future RCB implementation and policy development.

All included records were read in full by the lead reviewer (TGH), who undertook the initial preliminary extraction. Extracted data were grouped into categories and mapped against the SEM to provide a multi-level lens of influences on health workforce attraction and retention. Themes were organised across individual-level factors (e.g., skill, knowledge, and confidence), organisational-level factors (e.g., leadership, research culture, protected time, and peer support), and system-level factors (e.g., policy incentives, inter-institutional collaboration, and structural funding mechanisms). This deductive coding frame was subsequently refined with inductive codes derived from the data. Coding and theme development were supported through weekly discussions between the reviewers (TGH and ME), with discrepancies resolved through joint interpretation. Quantitative data reported in the included studies (e.g., proportion of health professionals who participated in RCB, retention outcomes, or evaluation metrics) were summarised descriptively and incorporated narratively within the relevant themes to contextualise qualitative insights. This integration of qualitative and quantitative findings provided a richer understanding of how and why research engagement may influence workforce outcomes in rural and remote settings.

Consistent with JBI guidance for scoping reviews, no formal quality appraisal or risk of bias assessment was conducted, as the aim was to map the breadth and depth of available evidence rather than evaluate intervention effectiveness or calculate pooled effect estimates.

## Results

### Description of studies

**Fig. 1:**
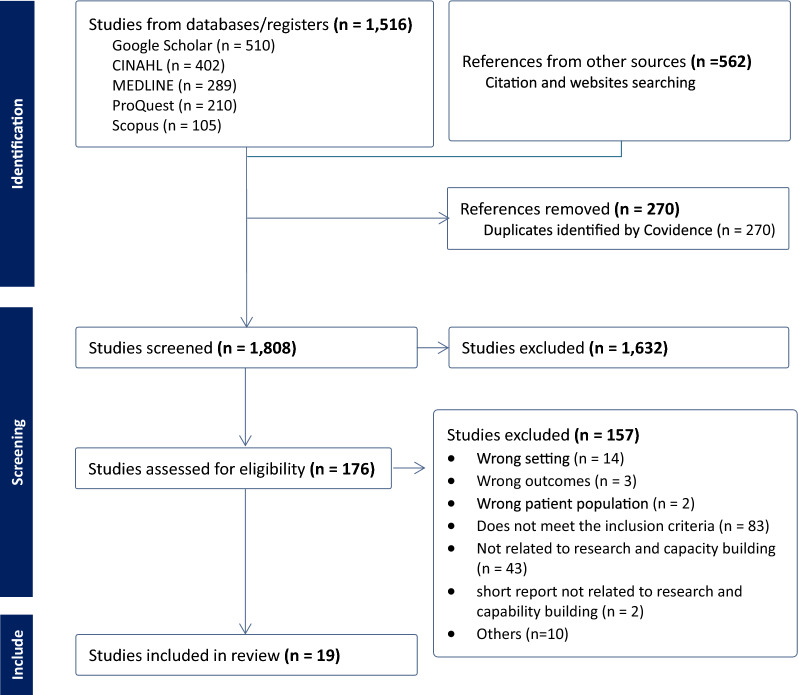
PRISMA flow diagram

A total of 2078 records were identified and 270 were removed for duplications. After screening 1808 titles and abstracts, 176 full texts were assessed for eligibility, and 19 studies met the inclusion criteria [11, 13–22, 26, 40–46] (Fig. 1).

Of the included studies, nine were qualitative [11, 14, 17, 19, 20, 26, 40, 43, 46], six were quantitative [15, 18, 41, 42, 44, 45], and four employed mixed methods [13, 16, 21, 22]. Most were conducted in Queensland, Australia, with others spanning Victoria, NSW, the Northern Territory (NT), and multi-state. Study outcomes assessed included RCB implementation, impact and/or effectiveness of RCB, sustainability of RCB, barriers and enablers of RCB implementation, workforce outcomes, and multiple outcomes (Table 1 and the details in supplementary Table S4).

**Table 1:** Description of the included studies (*n* = 19)

Variables	Categories	*n* (%)
Year of publication	Before 2015	4 (21.1)
	2015 onward	15 (78.9)
Study area/state	New South Wales	4 (21.1)
	Queensland	8 (42.0)
	Victoria	6 (31.6)
	Northern Territory	1 (5.3)
Study type	Primary study/peer-reviewed articles	17 (89.5)
	Report (government/admin report/reviews of documents)	2 (10.5)
Methods	Qualitative	9 (47.4)
	Quantitative	6 (31.6)
	Mixed methods	4 (21.0)
Health profession	Allied health professionals (pharmacy, physiotherapy, occupational therapy, …)	7 (36.8)
	Administrators (policy managers, …)	3 (15.8)
	Mixed professions (more than one category)	6 (31.6)
	Others*	1 (5.3)
	Not mentioned	2 (10.5)
Data collection method	Survey	7 (36.8)
	Qualitative interview(in-depth)	4 (21.0)
	Focused group discussion	1 (5.3)
	Multiple data collection methods**	7 (36.8)
Study outcome assessed	Implementation of RCB	3 (15.8)
	Effect/impact on EBP	3 (15.8)
	Barriers and facilitators of RCB implementation	2 (10.5)
	Multiple outcomes***	8 (42.1)
	Research capacity	2 (10.5)
	Effectiveness	1 (5.3)

*RCB* Research capacity building, *EBP* evidence-based practice

*Others included professionals other than the listed categories

**Multiple data collection methods—a study used more than one data collection method

***Multiple outcomes included studies with more than one outcome assessment

### Description of RCB

We provide an overview of the RCB initiatives identified in the included studies, summarising their aims and scope, key components and delivery approaches, and target professional groups.

Across the included studies, we found that research training and RCB initiatives have been implemented and/or evaluated in rural and remote healthcare areas in Australia, particularly in NSW [13, 17, 18, 26], Victoria [11, 19, 21, 22, 41, 43], and Queensland [14–16, 40, 42, 44–46]. Although the initiatives varied in scope and scale, they shared common aims, and several were embedded within health service structures, fostering a research culture and supporting evidence-informed practice [11, 26, 46]. The following subsections outline the aims, components, delivery approaches, and target professional groups of these RCB initiatives.

#### Aims and scope

RCB across rural health services in Australia was designed with overlapping but distinct aims and scopes. Most commonly, RCB aimed to strengthen individual research skills, build clinicians’ confidence in research engagement, and foster a supportive organisational research culture [19, 21, 43]. Some studies primarily focused on assessing research capacity and culture (RCC) among health professionals rather than directly strengthening individual RCB [15, 41, 42, 45], while others evaluated the outcome of research investment, including the introduction of dedicated research roles within rural health services. These were linked to enhanced clinician engagement in research, improvements in clinical practice, and workforce development benefits [14, 16, 17, 40, 46].

#### Program components and delivery approaches

RCB adopted varied components and delivery approaches tailored to rural and remote settings. Many studies identified structured training through short courses, research translation workshops, and postgraduate qualifications as the major components of RCB [13, 17, 19, 21]. Mentorship was central with models, including one-to-one supervision, group mentoring, and peer networks to support novice researchers and foster a culture of inquiry [13, 21]. Some programs used a decentralised, modular design with hub-based delivery, teleconferencing, and structured mentoring [18, 26].

We found that RCB delivery approaches were designed for flexibility, often incorporating hybrid, onsite, and offsite modes to accommodate geographic isolation and workforce constraints [19, 21, 43]. In many initiatives, dedicated research positions through ongoing education, supervision for higher degrees, and embedded mentoring within clinical teams were the forms of RCB delivery [14, 46]. Beyond individual and team-level interventions, some studies identified that focusing on broader system engagement, involving policy review, stakeholder consultation, and the development of strategic frameworks to embed/integrate research within organisational governance [11, 17, 20, 26, 40]. For example, some RRCBPs are embedded within health service structures, as seen in Queensland [46], NSW [26], and Victoria [11]. While other programs are developed and delivered through rural clinical schools, such as the program at Charles Darwin University in the NT [20].

#### Targeted professional groups

Most programs were profession-specific or designed for multidisciplinary participation, reflecting the diversity of rural and remote health workforces. Target groups included allied health professionals (e.g., physiotherapists, occupational therapists, and speech pathologists) [14, 15, 18, 21, 22, 26, 44]. Also included were nurses and midwives, general practitioners (GPs), Aboriginal health workers, community health practitioners, and supportive staff [11, 19, 26, 43]. Some programs specifically targeted early-career professionals and those returning to rural practice after training in metropolitan centres [46].

### Impact of RCBs on health workforce outcomes

**Fig. 2:**
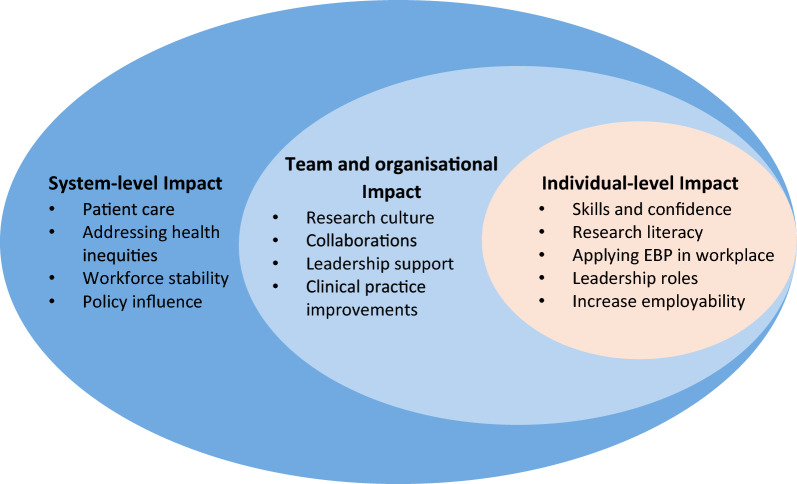
Multi-level impact of RCB based on the socioecological framework

The impact of RCB on the health workforce can be conceptualised across three inter-related levels: individual, organisational, and system, based on the SEM. This framework recognises that change occurs not only through individual skill development but also through supportive organisational environments and broader health system structures that sustain RCB (Fig. 2).

#### Individual-level impact

At the individual level, findings suggest a potential association between RCB and improved research literacy, technical skills, and confidence to engage with evidence-based practice (EBP) [15, 21]. Allied health professionals perceived that their research skills, confidence, and experience following participation in RCB were improved [14, 15, 44].

Included studies suggest that clinicians gained confidence to assume research leadership roles [17], and reported greater engagement, which also contributed to enhance job satisfaction [13]. Research training potentially enables clinicians to maintain strong research skills, confidence, and engagement over time, contributing to both personal and professional growth [26]. In a similar study, graduates of RRCBP in NSW also reported improved employability and career progression through their research experience and leadership roles.

#### Team and organisational-level impact

We found that studies suggest that RCB strengthens research culture, fosters collaboration and research, and improves healthcare priorities alignment [11, 16, 46]. RCB initiatives contributed to enhance interdisciplinary collaboration and organisational culture [11, 19]. Findings reported that RCB contribute to improve organisational change, including more positive staff attitudes and institutional support for research [17]. A decentralised model further improved RCB through regionally tailored support [18]. Long-term outcomes from RCBP in NSW revealed that graduates often became research champions in their teams, mentoring colleagues, enabling new projects, and raising the profile of research within their organisations [26].

#### System-level impact

RCB enhanced clinical practices and supported workforce stability [40]. Similarly, research initiatives driven by local health services produced tailored interventions that improved staff retention, strengthened system responsiveness, and integrated research into care delivery [11, 41, 43]. In some cases, RCB influenced health policy, and investments in research were linked to the development of new policy frameworks and communities of practice, fostering more collaborative health systems [16].

RCB addressed social determinants of health, contributing to reduced health inequities [20]. Clinician-led research not only improved local services but also informed regional models of care. Sustained RCB can drive real-world improvements in service delivery, influence policy and practice within health organisations, and provide broader system benefits, including supporting staff retention [26].

### RCB as a workforce attraction and retention strategy

**Fig. 3:**
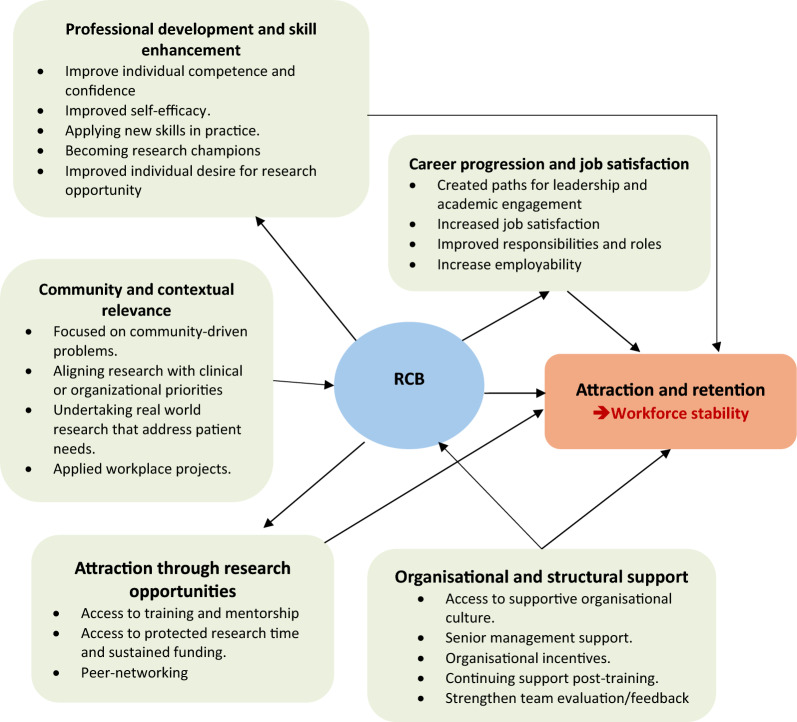
RCB as a workforce attraction and retention strategy. *RCB* research capacity building

Findings suggest that RCB is increasingly recognised as a strategy to strengthen workforce stability in rural and remote Australia [13, 14]. Although not primarily designed as workforce interventions, studies reported that RCB has been contributed to job satisfaction, professional development, and organisational support, all influencing workforce attraction and retention. The following subsections outline the mechanisms through which RCB contributes to these outcomes (Fig. 3).

#### Professional development and skill enhancement

RCB led to measurable improvements in research capacity among allied health professionals, with participants reporting moderate-to-high skill levels, with professional development identified as a major motivator [15]. Structured research training enhances practical research experience, such as idea generation, proposal development, and navigating research processes [44] while improving evidence-based practice knowledge, critical analysis skills, and research self-efficacy [19, 21, 22]. Training initiatives enhanced clinicians’ capacity to undertake research and increased professional fulfilment [13, 17]. Participants of RCBP reported enduring professional growth, describing the program as “transformative” in developing analytical thinking, project management, and communication skills [26], strengthening health staff professional identity and commitment to rural practice.

#### Retention through career progression and job satisfaction

Our findings suggest that research roles created pathways for leadership and academic engagement, which were associated with workforce stability [14, 46]. These roles supported clinician-led research, reinforced professional identity [16], and contributed to greater job satisfaction and development opportunities [15], all of which are key motivators for the health workforce.

Research engagement enabled early-career clinicians to take on new responsibilities and define clinician-researcher roles, which strengthened identity and commitment to rural practice [11, 43]. Participation in RCB increased clinicians’ confidence to assume research leadership, supporting retention through empowerment and recognition [17]. RCBP graduates attributed promotions, further postgraduate study, leadership roles, and renewed motivation to their research training [26].

#### Attraction through research opportunities

Government investment in research infrastructure and dedicated roles in Queensland attracted clinicians with strong research interests [14, 46]. Increased research experience gained through RCB further enhanced the appeal of rural practice [26, 44], while research opportunities were cited as a specific attraction mechanism among rural dietitians [42].

#### Retention through organisational and structural support

Findings showed that organisational support for research was contributed to recruitment success in South-Western Victoria, which was viewed as a marker of professional growth and opportunity [41].

Embedding research roles within clinical teams fostered a research-supportive culture that enhanced satisfaction, professional identity, and retention, particularly among allied health professionals [14, 46]. Opportunities to participate in research were valued for career development and the recruitment of health professionals seeking intellectually stimulating roles [11]. Organisational incentives, including recognition of training outcomes and alignment of research with career progression, further improved retention [19]. Studies suggest that retention gains depended on organisational support, noting that workplaces embedding research into clinical roles and recognising research achievements were contributed to retained skilled staff [26].

#### Retention through community and contextual relevance

RCB motivated clinicians to improve patient care using locally relevant evidence [41], while rural service-led and community-driven initiatives produced contextually appropriate interventions that strengthened workforce stability and aligned with local priorities [11, 20]. Tailoring research training and outputs to local health needs enhanced translation, professional satisfaction, and workforce outcomes [40, 43]. Embedding research within communities of practice and aligning it with clinical/organisational priorities promoted engagement, sense of ownership, and staff retention [14, 16, 19]. Contextual relevance remained critical for sustaining motivation, with staff undertaking ‘real-world’ projects that addressed local service needs and delivered tangible community benefits, reinforcing their commitment to rural practice [26].

### Enablers of RCB implementation and sustainability

**Fig. 4:**
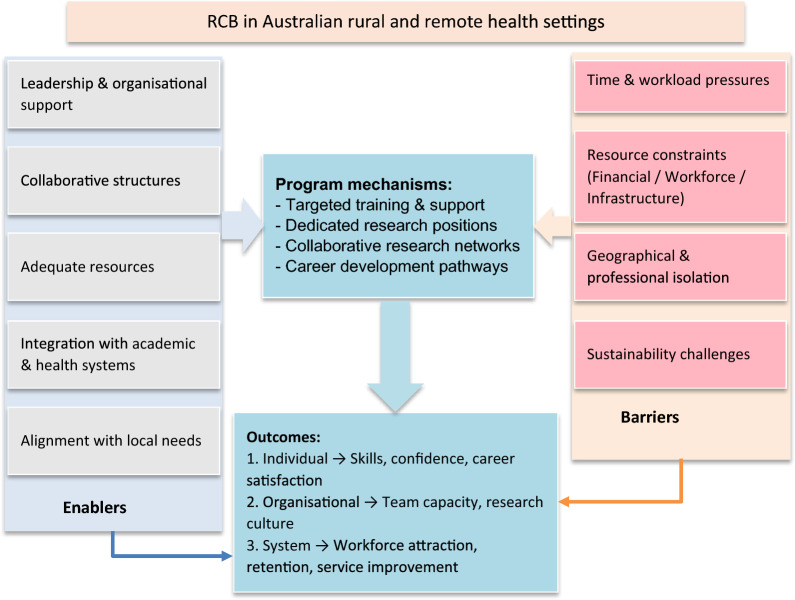
Enablers and barriers influencing RCB implementation, reach, and impact in the rural and remote Australian health system. *RCB* research capacity building

Evidence across studies highlighted several enablers that facilitated the successful implementation and sustainability of RCB in rural and remote settings, as outlined in the following subsections and summarised in Fig. 4.

#### Leadership and organisational support

The support from senior leaders, such as the Executive Director of Allied Health, was pivotal in establishing and sustaining dedicated research roles [14, 46]. Advocacy from health service leaders secured role clarity, embedded research into clinical responsibilities, and aligned research initiatives with broader organisational goals [16].

The senior leadership endorsement normalised clinician participation in research [11], further reinforced through recognition and incentives linked to training achievements [19]. The managerial commitment facilitated research integration into practice, improved attitudes toward research, and supported workforce development [17].

Leadership also played a critical role in collaborative research networks. Leaders with research expertise were better positioned to implement locally tailored strategies addressing rural needs [40]. Participation in inter-organisational partnerships legitimised research roles, enhanced local capability, and embedded research in routine service delivery [15, 18]. Participants of RCBP in NSW perceived that sustaining research momentum was difficult without senior management support, while supportive leaders helped them integrate research into practice and improve services [26].

#### Current collaborative structures

The university partnerships and internal networking were pivotal in establishing allied health research roles, offering mentorship, resources, and clearer pathways for clinicians [45, 46]. Partnerships with university researchers supported co-design and delivery of research training [11, 19], with longstanding partnerships shown to enhance research readiness [19].

Internal discipline-specific and cross-disciplinary engagement was also underpinned. This sustained research support was provided through shared learning, peer exchange, and project development [13].

Collaborative structures further supported translation into practice. Academic networks enabled knowledge mobilisation and translational pathways [43]. Strong linkages between researchers and primary care providers helped local RCB [20]. Likewise, the RCBP fostered an informal community of practice among its graduates, with participants often collaborating with peers and serving as research resources “research champions” for one another [26].

#### Current resource conditions

Protected research time, dedicated research positions, and supporting infrastructure (e.g., office space, supervision, and research committees) were essential for embedding allied health research roles [14] and supported continuity in decentralised programs [18]. Access to resources and facilities also strengthened the research culture [16]. Backfill availability and administrative support enabled clinician participation [13]. Furthermore, nationally, administrative support and sustained funding were emphasised as vital in resource-limited settings.

Unequal access to digital tools highlighted the importance of technological resources for program delivery [19]. Programs with adequate resource backing were more likely to produce research-engaged clinicians confident in applying inquiry to practice [15]. Continued resource allocation (time, funding, and infrastructure) beyond the initial training period is critical for maintaining newly developed research skills and is reported across the individual, organisational and system levels [26].

### Barriers of RCB implementation and its reach and impact

Several contextual and systemic barriers affect the implementation of RCB and its reach and impact in rural and remote health settings. A summary of the barriers to research engagement, and RCB implementation and its impact on health workforce outcomes in rural and remote areas are presented in Fig. 4.

#### Time and workload pressures

Limited protected time and heavy clinical workloads were the most consistently reported barriers to research engagement across different levels. Both clinical time demands and the absence of formal research roles restricted participation [14–16]. Competing clinical responsibilities and educational workloads further hindered uptake of training and ongoing engagement [19, 21, 22, 41]. Time constraints were repeatedly identified as the single greatest barrier to research participation, and even many years after completing RCBP rural clinicians continued to report that daily clinical pressures left little room for research [13, 26]. Nationally, high and unpredictable workloads further reduced capacity for sustained engagement. In many rural areas, clinicians were expected to manage high clinical loads, limiting their ability to maintain ongoing research involvement [13, 15, 18]. Cumulative stress of balancing clinical service delivery with research responsibilities was recognised as a key challenge, particularly in understaffed settings [18, 20].

#### Resource constraints

Inconsistent and insufficient funding was a core barrier across settings at the organisational and system levels. Restrictive and fragmented funding models, such as those underpinning the Rural Health Multidisciplinary Training (RHMT) and Primary Health Care Research, Evaluation and Development (PHCRED) programs, were considered unsustainable for long-term RCB [20, 40]. Research projects were frequently underfunded and unevenly distributed across regions [14, 16, 41, 46]. Funding shortfalls also limited access to backfill, travel, and accommodation for research [19]. Reliance on short-term contracts and external grants created further instability and undermined sustained engagement [11, 14]. Even where interest and funding existed, workforce shortages and staff turnover significantly constrained research participation and also reduced institutional capacity [20, 21, 46]. Lack of dedicated research positions or formalised research career paths meant rural clinicians often depended on personal initiative to conduct research, which many found unsustainable alongside their regular duties [16, 26].

Rural clinicians also faced physical and digital infrastructure inadequacy. Limited internet connectivity, lack of access to libraries, and inadequate management systems hindered participation [11, 18, 19]. In some settings, research spaces and equipment were unavailable or repurposed for clinical use due to resource pressures [14]. Gaps existed between organisational commitment to research and the reality of inconsistent support and limited infrastructure, resulting in an over-reliance on individual initiative to create research opportunities [26].

#### Geographical and professional isolation

Geographical and professional isolation were major barriers to research engagement and RCB implementations. Health professionals frequently reported limited access to research networks, mentors, and peer feedback [11, 14, 22]. Distance from research hubs restricted collaboration and professional development, often compounded by poor coordination across sites [13, 18]. Outreach service delivery and travel demands further limited research engagement [45]. The absence of mentorship constrained the research capacity of early-career clinicians [18]. Rural health professionals were also frequently excluded from collaborative research networks, reducing opportunities for professional growth and career advancement [17]. Without ongoing mentorship or connection to a research community, many clinicians reported feeling isolated in their research efforts, which further diminished their confidence and motivation to continue [11, 26]. In large rural health services, the absence of structured support was a source of instability and contributed to increasing disengagement among staff [46].

### Strategies to enhance RCB implementation and health workforce outcomes

We identified numerous strategies to strengthen research engagement and RCB implementation and its contribution to health workforce outcomes.

#### Embedding dedicated research positions

Embedding dedicated research roles within clinical teams is a critical strategy for sustainable RCB and improving workforce outcomes. These positions, often funded by health services or training programs, provide local leadership, mentorship, and a visible research presence that normalises research as part of everyday clinical practice [11, 14, 16, 46]. For example, studies reported that Allied health research roles improved engagement and retention while addressing workforce inequities in under-resourced areas [14, 16, 46].

Ongoing opportunities, such as embedded research trainers and dedicated personnel, can sustain RCB and reduce geographical and professional isolation [11, 19]. One-off training initiatives were often insufficient unless accompanied by continued supporting roles or structured opportunities to apply research skills within the workplace [26]. In addition, adequate research space, time allocation and institutional recognition were considered essential to sustain meaningful engagement [18, 40].

#### Targeted training and support

Tailored training programs, from introductory workshops to postgraduate qualifications, were widely recommended to strengthen skills across disciplines and career stages [13, 22]. Flexible delivery, including face-to-face, virtual, and hybrid, modular formats, as well as co-design through regionally focused training aligned with workforce needs, were particularly important to maximise accessibility and contextual relevance in rural and remote settings [18, 19, 21, 26, 41, 43].

Mentoring, both structured and informal, was a central sustaining research engagement and RCB, with one-to-one support improving confidence and skills, as well as further contributing to retention and professional development [13]. Targeted education and mentoring increased research activity and engagement, particularly when tailored to specific tasks [16, 45, 46]. Formal education alone, without opportunities for practical application or organisational support, may have limited long-term benefits [26].

#### Aligning research with local needs

Aligning research with health service goals, tailoring research to context and priorities increased institutional support and clinician participation [11, 43]. Systematic translation support and alignment with organisational goals were critical for translation into actionable outcomes [16, 40]. Addressing social determinants of health was crucial for ensuring that research met community needs and contributed to equity goals [20]. Co-designing research with rural health professionals was cited as a key strategy for making training and research contextually relevant [19]. In addition, encouraging “close-to-practice” research promotes engagement and RCB, as locally relevant projects are more likely to yield benefits to communities and tangible service improvements [26].

#### Establishing and expanding collaborative networks

Formalised networks helped ensure that rural research-informed policy and practice [43], while partnerships with universities enhanced local research relevance and impact [11]. Sustained university–health service partnerships can provide mentorship, collaboration, and access to resources [14].

Cross-site networking fostered shared learning and collaborative research across geographically dispersed sites [18, 26]. For instance, alumni of RCBP who remained connected reported ongoing collaboration and encouragement to continue research, and served as research champions within their local health districts [26]. In addition, a partnership between researchers, clinicians, and health providers ensured that research is aligned with community needs [20].

#### Securing sustainable funding and resources

Strengthening funding mechanisms and administrative support was essential to enhance and maintain RCB [15]. Departmental or organisational funding supported research activities and promoted workforce participation [42]. Targeted funding for specific research activities, such as proposal writing, helped reduce participation barriers for rural health professionals [45]. Aligning funding priorities with clinical needs was especially critical in under-resourced settings [16].

Addressing infrastructure gaps and resource shortages was also recommended to improve RCB among health professionals [41]. Extended support beyond initial training, including dedicated post-RCBP research positions, could maximise the return on RCB investment and assist in retaining experienced health staff [26].

## Discussion

### Main findings

Our scoping review mapped the available evidence on research engagement and RCB and the association with health workforce outcomes, including attraction and retention, in rural and remote areas in Australia. Although evidence was unevenly distributed across states and territories, findings consistently indicated that, despite varying in scope, structure, and delivery, RCB initiatives shared a core objective of strengthening research capacity at individual, organisational, and system levels. Few findings reported that RCB initiatives were often embedded within rural health services, linked to academic institutions, or led by the state/territory government, supported by diverse stakeholders, and designed for multidisciplinary participation. While not primarily established as workforce strategies, findings suggest a potential association between RCB and improved job satisfaction, professional development, and career progression, thereby contributing to workforce stability. Key enablers of successful RCB implementation and its impact on health workforce outcomes included supportive leadership, collaborative networks, and access to resources, while barriers such as time and resource constraints, limited infrastructure and technology, and professional and geographical isolation were frequently reported. Strategic approaches, such as embedding dedicated research roles, aligning research with local health service priorities, and securing sustainable funding, emerged as critical to advancing RCB implementation and enhancing its contribution to health workforce outcomes, including retention, attraction and stability in rural and remote settings.

### Interpretation of the findings

Our review highlights that RCB is vital and multifaceted in Australia’s rural and remote healthcare systems. These programs embed research into routine clinical work and community practice, which is important in settings, where workforce shortages, limited infrastructure, and complex local health needs demand locally grounded solutions.

By aligning research with community and clinical priorities, RCB generates contextually relevant, practical evidence that could enhance both patient care and workforce outcomes, including stability [14, 15, 20, 26, 43, 46]. A consistent finding was reported that RCB initiatives are most successful when tailored to local needs and supported by committed leadership. In a similar report, embedding programs within service frameworks encourages participation by a wide range of clinicians, including nurses and allied health professionals, to participate meaningfully in research [15, 46]. This engagement extends beyond skill development; it strengthens professional identity, deepens commitment to rural practice, and supports staff retention [16, 40]. By promoting a sense of belonging and professional recognition, RCB contributes to enhance job satisfaction and contributes to long-term workforce stability.

The effectiveness of research training, however, depends on program structure, institutional support, and adequate resources [11, 40]. Where these are lacking, clinicians’ participation declines, highlighting the importance of adaptable, context-specific approaches rather than universal models. Importantly, RCB influences rural healthcare systems at multiple levels, building individual confidence and research skills, promoting team collaboration and shared learning, and strengthening partnerships with academic institutions. This systemic impact reinforces that RCB would extend beyond isolated training to foster networks and cultures of continuous inquiry [11, 14, 19, 45, 46]. Sustained, collaborative engagement strengthens professional connections, reduces isolation, and enhances organisational culture, supporting staff attraction and retention. Ultimately, when RCB initiatives are well-resourced, contextually relevant, and integrated into routine clinical operations, clinician engagement is broad, sustained, and extends across professional and geographic boundaries. Investment in RCB could be recognised as a strategic priority for strengthening the rural and remote health workforce, improving service quality, and enhancing health outcomes for rural communities [13, 14, 46]. Evidence suggests that well-designed RCBPs also contribute to create rewarding and sustainable career pathways, encouraging clinicians to join and remain in rural and remote healthcare practice.

### Comparison with existing literature

Our findings are consistent with previous evidence from other developed countries, which shows that research engagement among rural clinicians contributes to greater job satisfaction, improved health service, and enhanced workforce stability [26, 47–50]. RCB has been described as a mechanism for fostering professional development, reducing professional isolation, and enabling context-specific innovations [12, 47]. Previous empirical studies from Australia highlighted the importance of leadership, mentorship access, and integration of RCB within health systems as key enablers of success [51, 52]. Similarly, studies from Canada underscore the role of context, including local populations, organisational research culture, provincial health systems, and funding structures, in shaping RCB success [53]. Also, identified were strategies for embedding a culture of research, such as communication, relationship building, mentorship, and training opportunities [54]. Evidence from the UK further shows that greater research activity and engagement by healthcare providers are associated with improved patient outcomes [55]. Collectively, these underscore that RCB can extend beyond generating research outputs.

This study supports and extends this body of evidence by offering a national perspective, including multiple health professions, jurisdictions, and program models. It illustrates how research engagement and RCB operate across various levels: supporting individual professional identity, enabling team-based collaboration, and strengthening system-level academic–clinical partnerships. These findings also align with Cooke’s research capacity-building framework, emphasising multi-level, sustainable, and contextually embedded approaches [56].

Importantly, this study contributes valuable insights into the sustainability of rural and remote health workforce through RCB. While previous studies have primarily focused on short-term skill development [52, 57], our findings showed how well-supported programs can promote long-term workforce retention by aligning professional development with health service priorities and enhancing career satisfaction, particularly in underserved areas [15, 18, 40]. This long-term alignment between RCB participation and career pathways reduces staff turnover and promotes continuity of rural practice, thereby strengthening rural and remote health service stability. Our finding is especially significant for Indigenous health, where culturally responsive research engagement supports workforce stability and fosters community trust. Although few studies have focused specifically on the research capacity of Aboriginal and Torres Strait Islander (ATSI) health professionals, existing reviews highlight the broader benefits of research engagement in workforce development and health system improvement [51].

### Implications of the findings

Our findings underscore the potential of RCB to be used not only as educational or quality improvement initiatives, but as strategic workforce interventions in rural and remote healthcare systems [26, 47]. Embedding dedicated research roles across all health professions, including under-represented groups, such as GPs, medical doctors, and Aboriginal Health Workers, can support career progression, strengthen professional identity, and improve workforce retention in underserved settings. In addition, providing clear research pathways and academic partnerships may enhance the appeal of rural and remote health services to early-career professionals, thereby contributing to staff attraction and retention.

At the policy and system level, RCB contributes to long-term workforce stability by ensuring ongoing professional support. National and jurisdictional frameworks might be benefited by embedding RCB within rural health workforce strategies, supported by sustained investment/funding models and cross-sector partnerships among health services, academia, and communities. Such partnerships are particularly appealing to clinicians considering rural practice. Incentivising collaborations between academic institutions and rural health services has the potential to promote knowledge exchange and workforce development. Ensuring that research priorities in policy guidelines align with community needs could enhance RCB implementation and its impact on health workforce outcomes.

At the organisational level, research engagement and RCB can strengthen rural and remote health services through contributing to evidence-based resource allocations and fostering team-based research cultures, which are vital for better workforce outcomes and stability. Effective strategies, such as formal mentorship schemes, visible career pathways, and participation in collaborative networks at the organisational level, can further improve staff attraction and retention. Embedding structural enablers, such as protected research time, dedicated research positions, sustained mentorship, and access to research infrastructure, within rural health workforce planning could support long-term organisational sustainability. When clinicians are supported to pursue research, they are more likely to view their workplace as professionally rewarding, enhancing staff retention and attraction.

At the individual level, research engagement and RCB foster professional growth, enhance job satisfaction, and empower clinicians, particularly those in isolated contexts, to lead locally relevant innovations. Participation in research provides professional challenge and recognition, key motivators for remaining in rural roles. However, ensuring sustained access to research opportunities in rural and remote settings requires continuing and tailored support. In programs, prioritising flexibility, context-specific training, underpinned by strong local and external networks, and co-design with frontline staff maximises relevance, engagement, and uptake.

Evidence gaps remain on the sustained impact of research engagement and RCB beyond short-term skill acquisition. Adopting longitudinal and implementation-focused study designs in future research would be valuable to assess the impact of RCB on career development, workforce retention and attraction, and, furthermore, on clinical practice and community health outcomes [50, 58]. Research priorities include under-studied Australian states and territories, such as NT and WA, as well as specific professional groups, including GPs, hospital-based medical officers, and Aboriginal Health Workers. In addition, there is a need for comparative evaluations across jurisdictions to identify context-specific and transferable strategies, and to better understand how contextual factors influence RCB implementation and its impact on the health workforce.

### Strengths and limitations of the study

A key strength of our scoping review was the use of best-practice methodological checklists and a comprehensive search strategy that drew on a wider range of databases and search engines. This approach allowed us to map the most recent evidence on RCB and its association with health workforce attraction and retention in rural and remote Australia. In addition, our review provided robust insights into how RCB influences workforce outcomes, identified key enablers and barriers for successful RCB implementation, and highlighted strategies relevant to future RCB policy and practice. The inclusion of studies employing diverse methodologies (qualitative, quantitative, and mixed methods) further strengthened the comprehensiveness of our synthesis and enhanced its practical relevance.

Despite these strengths, we acknowledged some limitations. First, the relatively small number of studies included may limit the generalisability of our findings. Although we covered a broad range of RCB studies across Australia, our scope was constrained by our reliance on published literature and publicly available grey literature. Publicly unavailable unpublished or internal evaluation reports, which could provide additional insights, were excluded, potentially resulting in an incomplete understanding of the full scope and effectiveness of RCB. Second, the focus on Australian literature may reduce the applicability of our findings to other countries or health systems. However, given the context-specific nature of RCB, our findings may still offer transferable insights to countries with similar rural health challenges. Third, heterogeneity in study designs and outcome measures limited the depth of comparative synthesis. Variability in program reporting, with some studies lacking robust evaluation frameworks or clearly defined measures, hindered direct comparisons and deeper synthesis, particularly in assessing effectiveness and identifying best practices. Fourth, another possible limitation might be publication bias. Finally, several areas remain under-researched, including ASTI health workers, medical/GP professions, and other jurisdictions (e.g., Tasmania, NT, and WA), which are often under-represented in broader policy discussions. As such, our findings should be interpreted with caution, particularly regarding generalisability and long-term sustainability, as well as the scalability of RCB initiatives.

## Conclusions

Our scoping review explored the role of RCB in health workforce outcomes in Australian rural health settings, focusing on its impact on workforce attraction and retention. The findings consistently demonstrate that although the design and delivery of RCB initiatives vary, they share a core objective: strengthening research capacity at individual, organisational, and system levels. By embedding research within rural health services, linking with academic institutions, or being implemented by the State and Territory governments, findings suggest that RCB contributes to job satisfaction, professional development, and career progression, ultimately supporting health workforce stability. Our review achieved its aim of synthesising existing evidence and identifying key enablers and barriers of RCB in rural and remote health settings. Successful programs were supported by strong leadership, collaborative networks, and adequate resources, while barriers such as time constraints, limited resources, and professional isolation were common. Key strategies to enhance the effectiveness of RCB include embedding dedicated research roles, aligning research with service priorities, and ensuring sustainable funding. It is essential to integrate research roles within broader health workforce strategies and ensure accessible mentorship and research infrastructure. Aligning research agendas with local community needs can further strengthen the impact of RCB on health workforce. Future research should evaluate the long-term effects of research engagement and RCB on workforce outcomes, including on retention and attraction, particularly in remote and rural communities across diverse health professions. In addition, longitudinal studies are necessary to assess the sustainability of RCB across different settings.

## Supplementary Information

Additional file 1.

Additional file 2.

Additional file 3.

Additional file 4.

Additional file 5.

## Data Availability

All data supporting the findings of this study are included in the manuscript and its supplementary materials.

## Abbreviations

AIHW: Australian Institute of Health and Welfare
ATSI: Aboriginal and Torres Strait Islander
DALYs: Disability adjusted life years
EBP: Evidence-based practice
FTE: Full-time equivalent
GP: General practice
HETI: Health Education and Training Institute
MRFF: Medical research future fund
NSW: New South Wales
NT: Northern Territory
PHCRED: Primary Health Care Research, Evaluation and Development
RCB: Research Capacity Building
RCBP: Research Capacity-Building program
RHMT: Rural Health Multidisciplinary Training
RRCBP: Rural Research Capacity-Building programs
RRR-Cap: Rural and Remote Research Capacity-Building program
SEM: Socioecological Model
WA: Western Australia
