# Supplementary material for: A scoping review of research capability building: impact on health workforce attraction and retention in rural and remote Australia
Source: Hum Resour Health. 2026 Apr 25;24:25. doi: 10.1186/s12960-026-01069-9 (PMC13262444; doi:10.1186/s12960-026-01069-9)
Supplement: Supplementary file 2 — Additional file 2. [file 12960_2026_1069_MOESM2_ESM.docx]

**Supplementary Table S1.** Definition of terms.

| **Terms** | **Definition** |
| --- | --- |
| *Research Capacity building* | The process of developing the abilities, skills, resources, and infrastructure of health professionals and healthcare facilities to conduct high-quality research in rural and remote areas. |
| *Research culture* | The shared values, attitudes, and norms that encourage, support, and prioritise research within rural and remote health facilities(Canti, Chrzanowska et al. 2021). Research culture can be an outcome of RCB, as one of its aims is to foster strong research and EBP culture at the individual, organisational, and system levels. |
| *Research development*: | The process of strategically planning and enhancing research activities, structures, and opportunities to improve research quality and output in rural and remote areas. |
| *Research engagement* | The participation of health professionals and health facilities in research activities, which may include planning, conducting, disseminating, or applying research in rural and remote areas. |
| *Scoping review* | A type of review used to assess the potential size and scope of the available research literature, aiming to identify the nature and extent of evidence on RCB and its contribution to health workforce outcomes(Grant and Booth 2009). It used to know the breadth and depth of evidence on a specific area for further research. |
| *Socioecological Model (SEM)* | A theoretical research framework used to examine how RCB influences workforce outcomes across multiple levels, providing a critical perspective on the complex drivers of workforce outcomes in rural and remote settings, and how factors at different levels interact to affect RCB (McLeroy, Bibeau et al. 1988). |

**Reference**

Canti, L., A. Chrzanowska, M. G. Doglio, L. Martina and T. Van Den Bossche (2021). "Research culture: science from bench to society." Biol Open **10**(8).

Grant, M. J. and A. Booth (2009). "A typology of reviews: an analysis of 14 review types and associated methodologies." Health Information & Libraries Journal **26**(2): 91-108.

McLeroy, K. R., D. Bibeau, A. Steckler and K. Glanz (1988). "An ecological perspective on health promotion programs." Health education quarterly **15**(4): 351-377.
