# Supplementary material for: A scoping review of research capability building: impact on health workforce attraction and retention in rural and remote Australia
Source: Hum Resour Health. 2026 Apr 25;24:25. doi: 10.1186/s12960-026-01069-9 (PMC13262444; doi:10.1186/s12960-026-01069-9)
Supplement: Supplementary file 3 — Additional file 3. [file 12960_2026_1069_MOESM3_ESM.docx]

**Supplementary Table S2:** Search strategies and search results for a scoping review of RCB and its impact on health workforce outcomes in rural and remote Australia.

| # | Search strategy | Results |
| --- | --- | --- |
| Medline/Ovid | | |
| #1 | ((("health care" or healthcare or health or medical or nurse) adj2 (personnel or professional* or provider* or staff or worker* or manpower)) or ("human resource*" or Internist or doctors or "allied health" or audiologist or "dental staff" or dentist or dietician) or ((language or speech) adj2 (pathologist or therapist)) or (midwi* or nurse or nutritionist or "occupational therapist*" or ophthalmologist* or p?diatrician or pharmacist* or physician or "general practitioners" or surgeon or radiologist or radiographer or psychologist or physiotherapist or "physical therapist")).ti,ab. | 1,059,047 |
| #2 | exp Health Personnel/ OR exp Workforce/ OR exp Health Workforce/ OR exp Physicians/ OR exp Allied Health Personnel/ OR exp Allied Health Occupations/ OR  exp Nurses/ OR exp Health Occupations/ | 2,396,384 |
| #3 | #1 OR #2 | 3,058,420 |
| #4 | ((Capability OR Capacity) adj2 (building OR development)).ti,ab OR ((Research) adj2 (activities OR collaboration OR engagement OR initiatives OR involvement OR mentorship OR network OR participation OR partnership OR project OR programs OR skills OR support OR training)).ti,ab OR ((action OR participatory) adj (research)).ti,ab OR ("analytical skills" OR "programme evaluation").ti,ab OR ((knowledge) adj2 ("utilisation research" OR building OR transfer)).ti,ab OR ((scientific) adj2 (evidence OR knowledge OR Skill)).ti,ab | 124,154 |
| #5 | exp Capacity Building/ OR exp Mentoring/ OR exp Mentors/ OR exp Research/ OR exp Health Services Research/ OR exp Program Evaluation/ OR exp Evidence-Based Practice/ OR exp Evidence-Based Medicine/ OR exp Translational Research, Biomedical/ OR exp Nursing Research/ | 1,000,668 |
| #6 | #4 OR #5 | 1,092,022 |
| #7 | ((employee OR employment OR staff OR talent) adj2 (attraction OR benefits OR engagement OR loyalty OR reward OR retain OR retention OR recruitment OR turnover OR well-being OR "work-life balance")).ti,ab OR ((organisational OR organizational) adj2 (change OR characteristic* OR culture OR determination OR innovation OR process OR support OR commitment OR impact OR outcome)).ti,ab OR ((workforce OR work OR job) adj2 (development OR engagement OR influence OR motivation OR satisfaction OR security OR stability)).ti,ab OR ("intention to leave").ti,ab | 43,931 |
| #8 | Job Satisfaction/ OR Personnel Management/ OR Personnel Turnover/ OR exp Organizational Innovation/ OR Organizational Culture/ OR exp Staff Development/ OR exp Burnout, Professional/ OR exp Job Security/ OR exp Efficiency, Organizational/ OR exp Workplace/ OR exp employment/ | 218,792 |
| #9 | #7 OR #8 | 244,309 |
| #10 | (Australia* OR Canberra OR "new south Wales" OR Sydney OR Victoria OR Melbourne OR Queensland OR Brisbane OR Adelaide OR Tasmania OR Hobart OR Perth OR "western Australia " OR Darwin OR "northern territory").ti,ab | 214,284 |
| #11 | exp Australia/ OR exp Australian capital territory/ OR exp new south Wales/ OR exp northern territory/ OR exp Queensland/ OR exp south Australia/ OR exp Tasmania/ OR exp Victoria/ OR exp western Australia/ | 177,642 |
| #12 | #10 OR #11 | 266,390 |
| #13 | ((Country OR countryside OR nonmetropolitan) adj (healthcare OR "health care" OR "health services" OR "health facilities" OR "medical service" OR "health sector")).ti,ab OR ((deprived OR disadvantaged) adj (area OR community OR groups OR province OR region)).ti,ab OR ((remote OR rural) adj (area OR clinic OR community OR health OR "health care" OR healthcare OR "health service*" OR hospitals OR "medical care" OR "medical services" OR province OR region OR settings OR population)).ti,ab OR ("shortage area").ti,ab OR ((unequal OR inequitable) adj (distribution)).ti,ab | 58,321 |
| #14 | exp Rural Health/ or exp Rural Health Services/ or exp Primary Health Care/ or exp Vulnerable Populations/ or exp Medically Underserved Area/ or exp Healthcare Disparities/ | 273,114 |
| #15 | #13 OR #14 | 318,029 |
| #16 | #3 AND #6 AND #9 AND #12 AND #15 | 300 |
| #17 | limit #16 to (humans and yr="2000 -Current") | 289 |
| CINAHL Ultimate/Ebsco | | |
| #1 | TI (("health care" OR healthcare OR health OR medical OR nurse) N2 (personnel OR professional* OR provider* OR staff OR worker* OR manpower)) OR AB (("health care" OR healthcare OR health OR medical OR nurse) N2 (personnel OR professional* OR provider* OR staff OR worker* OR manpower)) OR TI ("human resource*" OR Internist OR doctors OR "allied health" OR audiologist OR "dental staff" OR dentist OR dietician) OR AB ("human resource*" OR Internist OR doctors OR "allied health" OR audiologist OR "dental staff" OR dentist OR dietician) OR TI ((language OR speech) N2 (pathologist OR therapist)) OR AB ((language OR speech) N2 (pathologist OR therapist)) OR TI (midwi* OR nurse OR nutritionist OR "occupational therapist*" OR ophthalmologist* OR p?diatrician OR pharmacist* OR physician OR "general practitioners" OR surgeon OR radiologist OR radiographer OR psychologist OR physiotherapist OR "physical therapist") OR AB(midwi* OR nurse OR nutritionist OR "occupational therapist*" OR ophthalmologist* OR p?diatrician OR pharmacist* OR physician OR "general practitioners" OR surgeon OR radiologist OR radiographer OR psychologist OR physiotherapist OR "physical therapist") | 911,833 |
| #2 | (MH "Health Personnel+") OR (MH "Rural Health Personnel+") OR (MH "Health Occupations+") OR (MH "Physicians+") OR (MH "Nurses+") | 1,488,673 |
| #3 | #1 OR #2 | 1,965,744 |
| #4 | TI((Capability OR Capacity) N (building OR development)) OR AB((Capability OR Capacity) N (building OR development)) OR TI ((Research) N2 (activities OR collaboration OR engagement OR initiatives OR involvement OR mentorship OR network OR participation OR partnership OR project OR programs OR skills OR support OR training)) OR AB ((Research) N2 (activities OR collaboration OR engagement OR initiatives OR involvement OR mentorship OR network OR participation OR partnership OR project OR programs OR skills OR support OR training)) OR TI((action OR participatory) N(research)) OR AB((action OR participatory) N(research)) OR TI("analytical skills" OR "programme evaluation") OR AB("analytical skills" OR "programme evaluation") OR TI((knowledge) N2 ("utilisation research" OR building OR transfer)) OR AB((knowledge) N ("utilisation research" OR building OR transfer)) OR ((scientific) N2 (evidence OR knowledge OR Skill)) OR ((scientific) N2 (evidence OR knowledge OR Skill)) | 67.767 |
| #5 | (MH "Research+") OR (MH "Research Support+") OR (MH "Action Research") OR (MH "Mentorship") OR (MH "Professional Practice, Research-Based+") OR (MH "Medical Practice, Research-Based") OR (MH "Nursing Practice, Research-Based") OR (MH "Physical Therapy Practice, Research-Based") OR (MH "Occupational Therapy Practice, Research-Based") | 3,440,743 |
| #6 | #4 OR #5 | 3,459,778 |
| #7 | TI((employee OR employment OR staff OR talent) N2 (attraction OR benefits OR engagement OR loyalty OR reward OR retain OR retention OR recruitment OR turnover OR well-being OR "work-life balance")) OR AB((employee OR employment OR staff OR talent) N2 (attraction OR benefits OR engagement OR loyalty OR reward OR retain OR retention OR recruitment OR turnover OR well-being OR "work-life balance")) OR TI((organisational OR organizational) N2 (change OR characteristic* OR culture OR determination OR innovation OR process OR support OR commitment OR impact OR outcome)) OR AB((organisational OR organizational) N2 (change OR characteristic* OR culture OR determination OR innovation OR process OR support OR commitment OR impact OR outcome)) OR TI((workforce OR work OR job) N2 (development OR engagement OR influence OR motivation OR satisfaction OR security OR stability)) OR AB((workforce OR work OR job) N2 (development OR engagement OR influence OR motivation OR satisfaction OR security OR stability))  OR TI("intention to leave") OR AB("intention to leave") | 40,390 |
| #8 | (MH "Personnel Retention") OR (MH "Stability+") OR (MH "Work Engagement+") OR (MH "Job Satisfaction+") OR (MH "Burnout, Professional+") OR (MH "Employment+") | 141,165 |
| #9 | #7 OR #8 | 166,351 |
| #10 | TI(Australia* OR Canberra OR "new south Wales" OR Sydney OR Victoria OR Melbourne OR Queensland OR Brisbane OR Adelaide OR Tasmania OR Hobart OR Perth OR "western Australia " OR Darwin OR "northern territory") OR AB(Australia* OR Canberra OR "new south Wales" OR Sydney OR Victoria OR Melbourne OR Queensland OR Brisbane OR Adelaide OR Tasmania OR Hobart OR Perth OR "western Australia " OR Darwin OR "northern territory") | 101,714 |
| #11 | (MH "Australia+") OR (MH "Australian Capital Territory") OR (MH "New South Wales") OR (MH "Northern Territory") OR (MH "Queensland") OR (MH "South Australia") OR (MH "Tasmania") OR (MH "Victoria") OR (MH "Western Australia") | 133,086 |
| #12 | #10 OR #11 | 165,300 |
| #13 | TI((Country OR countryside OR nonmetropolitan) N2 (healthcare OR "health care" OR "health services" OR "health facilities" OR "medical service" OR "health sector")) OR AB((Country OR countryside OR nonmetropolitan) N2 (healthcare OR "health care" OR "health services" OR "health facilities" OR "medical service" OR "health sector")) OR  TI((deprived OR disadvantaged) N (area OR community OR groups OR province OR region)) OR AB ((deprived OR disadvantaged) N2 (area OR community OR groups OR province OR region)) OR TI((remote OR rural) N2 (area OR clinic OR community OR health OR "health care" OR healthcare OR "health service*" OR hospitals OR "medical care" OR "medical services" OR province OR region OR settings OR population)) OR AB ((remote OR rural) N2 (area OR clinic OR community OR health OR "health care" OR healthcare OR "health service*" OR hospitals OR "medical care" OR "medical services" OR province OR region OR settings OR population)) OR TI ("shortage area") OR AB ("shortage area") OR TI((unequal OR inequitable) N (distribution)) OR AB((unequal OR inequitable) N (distribution)) | 52,460 |
| #14 | (MH "Hospitals, Rural") OR (MH "Rural Health") OR (MH "Rural Health Services") OR (MH "Services for Australian Rural and Remote Allied Health") OR (MH "Remote Area Nurses") OR (MH "Remote Area Nursing") OR (MH "Medically Underserved Area") OR (MH "Rural Areas") OR (MH "Medically Underserved") OR (MH "Personnel Shortage+") OR (MH "Nursing Shortage") OR (MH "Medication Shortage") OR (MH "Catchment Area (Health)") | 69,596 |
| #15 | #13 OR #14 | 101,318 |
| #16 | #3 AND #6 AND #9 AND #12 AND #15 | 458 |
| #17 | Limiters to #16- Publication Date: 20000101-20250225; Human | 402 |
|  | SCOPUS |  |
| 1 | TITLE-ABS-KEY ( ( "health workforce" OR "healthcare workforce" OR "health professional*" OR "health personnel" OR clinician* OR nurse* OR doctor* OR physician* OR "allied health" OR practitioner* ) AND ( "research capacity building" OR "research capacity" OR "research capability" OR "research engagement" OR "research participation" OR "research training" OR "research development" OR "research skills" OR "research culture" ) AND ( Australia* AND ( rural OR remote OR regional OR "rural health" OR "remote health" OR "rural workforce" OR "remote workforce" ) ) ) | 132 |
| 2 | Limited #1 to keyword: human | 105 |
| ProQuest Central | | |
| 1 | (TI,AB("health workforce" OR "healthcare workforce" OR "health professional*" OR clinician* OR nurse* OR doctor* OR physician* OR "allied health") OR SU("health personnel")) AND (TI,AB("research capacity building" OR "research capacity" OR "research capability" OR "research engagement" OR "research training" OR "research development") OR SU("research training")) AND (TI,AB(Australia* AND (rural OR remote OR regional OR "rural health" OR "remote health")) OR SU("rural health")) | 225 |
| 2 | Limit #1 to subject: human | 210 |
|  | Google scholar |  |
| 1 | ("research capacity building" OR "research capacity" OR "research capability" OR "research engagement") AND ("health workforce" OR "healthcare workforce" OR "health professional" OR nurse OR clinician OR doctor OR "allied health") AND (attraction OR retention OR motivation OR stability OR turnover OR recruit*) AND (rural OR remote OR regional) AND Australia AND (human OR patient OR "health workers") | 510 |
|  | Google and snowballing |  |
|  | Search for Google: "Research capacity building" AND “Health workforce outcomes” AND "rural and remote Australia" | 562 |
|  | Then snowballing |  |
