# Supplementary material for: A scoping review of research capability building: impact on health workforce attraction and retention in rural and remote Australia
Source: Hum Resour Health. 2026 Apr 25;24:25. doi: 10.1186/s12960-026-01069-9 (PMC13262444; doi:10.1186/s12960-026-01069-9)
Supplement: Supplementary file 4 — Additional file 4. [file 12960_2026_1069_MOESM4_ESM.docx]

**Supplementary Table S3**. Study inclusion and exclusion criteria.

| Criteria | Definition | Inclusion | Exclusion |
| --- | --- | --- | --- |
| Population | - Health professionals | All health professionals, according to the AIHW health workforce classifications[1], including   - - Nurses and midwives   - Medical practitioners   - Dental practitioners   - Allied health professionals   - Mental health professionals   - Pharmacists |  |
| Concept/ interventions | - The phenomena of interest in the reviews | - Engagement in research and research capability-building initiatives - RCB - Research development - Research mentoring and supervision - Research supportive incentives | - Not involved/engaged in research or research capability building initiatives//programs - Other professional development programs |
| Outcome | - Health professionals’ attraction and retention | - Attraction and retention - Intention to stay/leave and sustainability |  |
| Type of studies | - Types of articles/evidence included in the review | - All observational studies, published and publicly available grey literature - Qualitative, quantitative and mixed method studies | - Studies not relevant to the review questions, or missed one or two of the eligibility criteria - Letters and editorials - Conference papers - Reviews including narrative, systematic, and scoping |
| Context/ settings | - Rural settings | - Regional - Remote - Rural - Underserved - Hard to reach - Countryside - Studies with both rural and metro areas were included, and results for rural areas were extracted. | - Urban areas - Major cities |
| Country |  | - Studies conducted in Australia | - Studies conducted outside Australia |
| Limiters | Year | - From January 1, 2000, onwards | - Studies published before 2000 |
|  | Studies species | - Human studies | - Animal studies |

**References**

1. AIHW: **Health workforce. Canberra: Australian Institute of Health and Welfare, 2024 [cited 18/05/2025]. Available from:** [**https://www.aihw.gov.au/reports/workforce/health-workforce**](https://www.aihw.gov.au/reports/workforce/health-workforce)**. .** 2024.
