# Supplementary material for: A scoping review of research capability building: impact on health workforce attraction and retention in rural and remote Australia
Source: Hum Resour Health. 2026 Apr 25;24:25. doi: 10.1186/s12960-026-01069-9 (PMC13262444; doi:10.1186/s12960-026-01069-9)
Supplement: Supplementary file 5 — Additional file 5. [file 12960_2026_1069_MOESM5_ESM.docx]

**Supplementary Table S4**. Description of included studies (n=19)

| **Author (year)** | **Area/State** | **Study objectives** | **Study design/settings** | **Study type** | **Study participants/profession** | **Data collection methods** | **Study outcome assessed** |
| --- | --- | --- | --- | --- | --- | --- | --- |
| Barnett et al. (2005) | NSW | To identify ways to build research capacity within primary health. Specifically, it aimed to identify research support, education and training needs; processes to enhance research engagement; and associated barriers and enablers to research involvement at the clinician level. | Mixed methods consultation study using a combination of a one-page survey and a guided meeting format in a primary health setting in rural NSW | Mixed | Primary health care (PHC) clinicians working in the Area Health Service (AHS) or private settings, and non-government organisations involved in direct primary health care or representing clinicians | One-page survey (26 participants) and guided meeting format (108 participants in 17 meetings - mostly group, 3 with individual managers) | - Identify support needs, processes to enhance research engagement, and barriers and enablers to clinicians’ research involvement. - Interest levels in different modes of research support and research topics. |
| Brown et al. (2022) | Queensland | To evaluate the impact of research investment from 2008 to 2018 at Townsville Hospital and Health Service (THHS), a regional Hospital and Health Service (HHS) in Queensland, Australia. The evaluation also sought to identify contextual conditions that enable or hinder intended impacts. | A mixed-method realist-informed evaluation | Mixed | Allied health, Nursing, Medicine, Managerial, and Research support staff | - Interview - Data from secondary sources (document reviews) | - Research investments - Research impacts - Contextual conditions influencing impacts" |
| Crombie et al. (2021) | Victoria | Understand the research capacity and culture (RCC) of the regional allied health workforce over time (2014 vs 2018) and identify strategies. | Cross-sectional study; comparison over time. Setting: Regional health service (various clinical settings), Victoria. | Mixed | Allied Health Professionals (AHPs) from various disciplines. | Open-ended questions within an online survey (RCC Tool). | - Research capacity and culture (RCC) at individual, team, and organisation levels - Motivators and barriers to research: Changes over time. |
| Edelman et al. (2020) | Queensland | To inform a research impact evaluation at THHS by developing a programme theory showing how research investment is expected to have impact. | Qualitative study; realist-informed design involving programme theory development. Setting: Regional Australian Hospital and Health Service (THHS). | QUAL | Health service executives and senior employees (offering perspectives from nursing, allied health, medicine, and whole-of-organisation roles). | - Analysis of strategic documentation - Semi-structured, in-depth interviews. | - Expected research impacts (categories: enhanced research activity/capacity; improved clinical practice, workforce capability/stability; patient/population health). - Programme theory development. |
| Gill et al. (2019) | Victoria | Investigate research interest, capacity, and culture (RCC) in individuals, teams, and health organisations across south-western Victoria; identify barriers/facilitators. | Cross-sectional survey | QUAN | All staff (clinical: nursing, allied health, medical; mental health; medical science; admin/clerical; corporate). | survey | Research capacity and culture at individual, team, and organisation levels; Research interest/involvement; Motivators and barriers to research participation. |
| Grundy J et al. (2003) | Northern Territory | To answer the questions:  What is the existing capacity for research evaluation and quality assurance?  What are the opportunities for strengthening the implementation of research, evaluation and quality assurance at the primary level?  What are the barriers to implementation of research, evaluation and quality assurance at the primary health care level?  How can research and evaluation capacity be sustained? | Qualitative study using literature review, interviews, consultations, and workshops in the Northern Territory | QUAL | Mixed: Primary health care providers, researchers, administrators, Indigenous health workers, and remote area nurses. | - Literature review of national and international literature - Interviews, consultations and workshops were conducted over a three-month period in Alice Springs and Darwin. | Barriers to research implementation at the primary care level: opportunities for strengthening research capacity |
| Howard et al. (2013) | Queensland | Investigate the research capacity and the factors that contribute to it at the individual and department level among the nutrition and dietetics workforce in Queensland, Australia. | Cross-sectional | QUAN | Dietitians and nutritionists | survey | Research capacity among dietitians |
| King et al. (2022) | Victoria | The study aims are:  1. Measure the effect of the research training program on participants' knowledge, attitudes and practice regarding evidence-based practice.  2. Explore the outcomes and impacts (if any) of the research training program on participants' individual EBP and research activity.  3. Explore the factors that influenced the outcomes and impacts of the training program on individual EBP and research activity; and  4. Explore any potential influence of the research training program on research capacity within participants' health organisations. | Sequential mixed methods | Mixed | Allied health professionals | Semi-structured interviews | - Evidence-based practices, knowledge, attitudes, and practice were measured using 23 research items that a registered nurse or other health professional might encounter in clinical practice, including the utilisation and conduct of research. |
| King et al. (2023) | Victoria | To explore rural health stakeholders' perspectives on capability-building needs for emerging researchers to enable research translation into health practice. | Qualitative descriptive study; social constructionist paradigm. Setting: Rural/regional health services, universities, primary health network, Victoria. | QUAL | Emerging researchers (health professionals with little/no research training /experience), research mentors, health managers/team leaders. | Three online focus groups using a semi-structured interview guide. | - Perceived capability-building needs for research translation; Understanding of research translation, Support needs for emerging researchers in rural settings. |
| Matus et al. (2019) | Queensland | - To evaluate the current research capacity and culture among Allied Health Professionals working in a large regional health service using the Research Capacity and Culture tool   To identify key components influencing allied health research capacity and culture at the level of individuals, teams, and organisations. | Cross-sectional observational | QUAN | Allied Health Professionals | survey | - Research capacity and culture at the individual, team and organisation level |
| Pain et al. (2015) | Queensland | Identify if the research supports the need for rural HPs to be similar or different from those of regional city health practitioners. | Cross-sectional | QUAN | Health practitioners who were public sector employees | survey | - Research experience and support needs - Knowledge of research methods and factors that influenced research engagement and participation, and barriers and enablers to engaging with research. |
| Pain et al. (2018) | Queensland | To determine whether allied health professionals’ research experience has increased since 2011 (the time of the research capacity building initiative). | Cross-sectional | QUAN | Allied health professionals | survey | - Research experience following the intervention |
| Quilliam et al. (2023) | Victoria | To identify characteristics of the design and implementation of current research training for rural health professionals in Victoria, Australia, to inform a future model for rural health professional research capacity and capability building. | A qualitative descriptive study was undertaken. Key informants with extensive knowledge of research education and training in rural health services in Victoria were invited to participate in semi-structured telephone interviews. The setting was rural health services in Victoria, Australia. | QUAL | The participants included rural health managers, rural health academics/research coordinators, and university sector directors/managers. | - Semi-structured telephone interviews | - Identification of design and implementation characteristics of current research training for rural health professionals in Victoria |
| Schmidt et al. (2016) | NSW | Describe the approach used within the Research Capacity Building Program and evaluate its effectiveness in meeting its aims as a development program | Cross-sectional/Project report | QUAN | Nursing, allied health and population health | survey | - Approach and effectiveness of research capacity building programs |
| Schmidt et al. (2022) | NSW | To understand the impact of undertaking research training for RCB graduates, their careers and their organisations. | Qualitative using a critical realism | QUAL | Rural health workers including Nursing, Allied health and others | Focus group discussion and interviews | Research capacity impact at individual, team and organisation level |
| Webster E et al. (2011) | NSW | To gain a better understanding of the impacts of the research program from the perspective of candidates, their managers and mentors and assessed the value of the capacity building framework for achieving the aims of the research program | Qualitative design | QUAL | Managers and mentors. | Semi-structured interviews | Capacity building outcomes |
| Wenke et al. (2017) | Queensland | To explore the impact of government-funded allied health research positions on building research capacity within their organisational context and describe mechanisms that enable and/or hinder their impact. | Qualitative methodology informed by a realist approach; individual interviews and stakeholder focus groups in governmental health services | QUAL | Allied health professionals (AHPs) include occupational therapists, nutritionists/dietitians, exercise physiologists, physiotherapists, speech pathologists, psychologists, medical scientists, managers, clinicians, team leaders, and professional heads. | - Semi-structured interviews and four focus groups | Impact of research positions on building allied health research capacity, mechanisms enabling/hindering their impact, and key outcomes at individual, team/service, and organisational/community levels. |
| Wenke, et al. (2018 | Queensland | To illustrate the outcomes of a dedicated allied health (AH) research position within a large Queensland regional and rural health service. The secondary aim was to describe the enabling and hindering mechanisms of the success of the role. | Descriptive case study of the Darling Downs Hospital and Health Service (DDHHS), which delivers clinical services to approximately 300,000 people across 26 Darling Downs and South Burnett locations, spanning approximately 90,000 km² | QUAL | Different professional backgrounds, including physiotherapy, occupational therapy, oral health, and medical imaging. | - One-on-one semi-structured interviews with the EDAH and research fellow, as well as a focus group with stakeholders. - Document analysis of records of key performance indicators | Outcomes of a dedicated research position on allied health research capacity, and the mechanisms that hindered or facilitated these outcomes and the position's ongoing success. |
| Wong Shee et al. (2022) | Victoria | To determine the contextual factors influencing research and re-search capacity building in rural health settings. The aim of this study was to describe the context of implementation and the contextual factors influencing RCB in rural health services. | Qualitative study using semi-structured telephone interviews. The setting was Victorian rural health services and university campuses. | QUAL | Senior rural health managers, academics, research coordinators, and university senior managers/directors. | - Semi-structured individual telephone interviews. | Main outcome measures: Contextual factors influencing the operationalisation and prioritisation of research capacity building in rural health services. The findings illustrated the implementation context and the complex contextual tensions, which either prevent or enhance research capacity building in rural health services. |
